# Myeloid Sarcoma Mimicking Intramuscular Abscesses in a Patient With Myelodysplastic Syndrome: A Diagnostic Challenge

**DOI:** 10.7759/cureus.93888

**Published:** 2025-10-05

**Authors:** Margarida Mourato, Marta Machado, Miguel Achega, Rita Penaforte, Catarina Negrão

**Affiliations:** 1 Internal Medicine, Hospital Professor Doutor Fernando Fonseca, Amadora, PRT

**Keywords:** extramedullary leukemia, intramuscular abscess, myelodysplastic syndrome, myeloid sarcoma, soft tissue mass

## Abstract

Myeloid sarcoma (MS) is a rare extramedullary tumor composed of immature myeloid cells. It typically occurs in the context of acute myeloid leukemia (AML) but may also present alongside myelodysplastic syndromes (MDS) and other myeloid neoplasms. We present the case of a 63-year-old male with no prior hematologic disease who was admitted with right lower limb swelling, initially diagnosed as cellulitis. During hospitalization, he developed multiple soft tissue lesions and progressive normocytic anemia. CT imaging showed multiloculated intramuscular collections. Despite negative blood cultures, methicillin-sensitive *Staphylococcus aureus *(MSSA) was isolated from drained material. The patient’s anemia and emerging neutropenia prompted bone marrow evaluation, revealing trilinear dyshematopoiesis consistent with MDS. Magnetic resonance imaging (MRI) and positron emission tomography-computed tomography (PET-CT) identified persistent abscess-like lesions without clear evidence of neoplasia. A muscle biopsy from the thigh confirmed MS. The disease later progressed to acute myelomonocytic leukemia with pancytopenia. This case underscores the importance of maintaining a high index of suspicion for MS in patients with atypical soft tissue lesions and unexplained cytopenias, particularly in the absence of overt leukemia. Prompt biopsy and immunophenotyping are essential for accurate diagnosis.

## Introduction

Myeloid sarcoma (MS) is a malignant extramedullary tumor composed of immature myeloid precursor cells, typically myeloblasts. It is most frequently associated with acute myeloid leukemia (AML), although it may also occur in patients with myelodysplastic syndromes (MDS), chronic myeloid leukemia (CML), or myeloproliferative neoplasms (MPNs) [1-3]. MS may present before, concurrently with, or after the diagnosis of systemic disease. Its estimated incidence is approximately 2-8% among patients with AML [2,4]. MS can occur in a wide range of anatomical locations, including the skin, bones, lymph nodes, central nervous system, gastrointestinal tract, and soft tissues. Due to its nonspecific presentation, with clinical and radiological features overlapping with more common conditions, MS is very frequently misdiagnosed as infection, lymphoma, or other soft tissue tumors [3,5,6]. Herein, we describe a case of MS presenting as presumed soft tissue abscesses in a patient ultimately diagnosed with MDS, highlighting the diagnostic challenges and the critical importance of biopsy in ambiguous clinical scenarios.

## Case presentation

**Figure 1:**
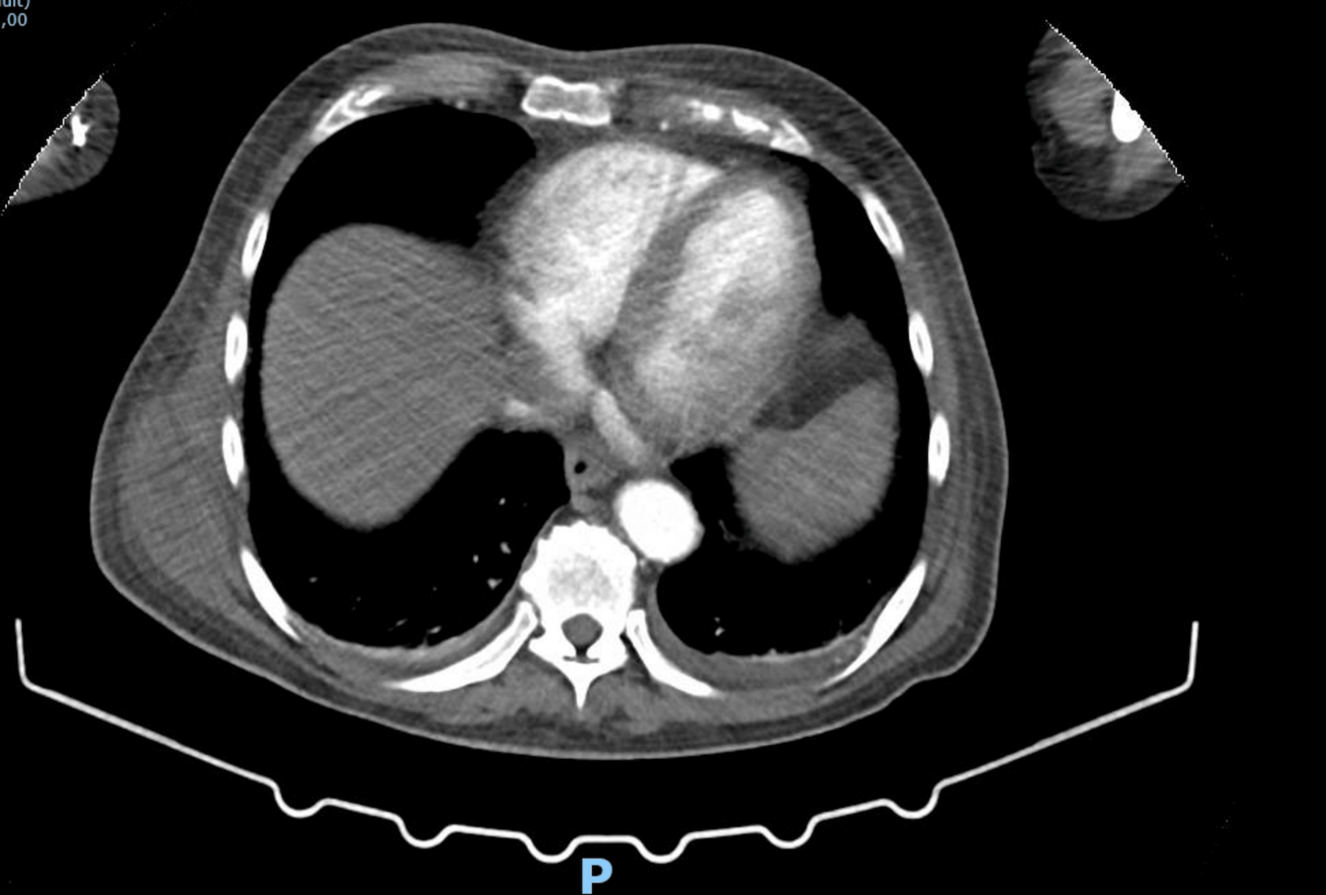
CT scan revealing abscess of the right latissimus dorsi muscle CT scan revealing an heterogenous area on the lateral aspect of the right latissimus dorsi muscle, located in the lateral region of the lower third of the right hemithorax, measuring approximately 63 x 35 mm with peripheral enhancement after intravenous contrast administration (abscess or infected hematoma) without evidence of active bleeding.

**Figure 2:**
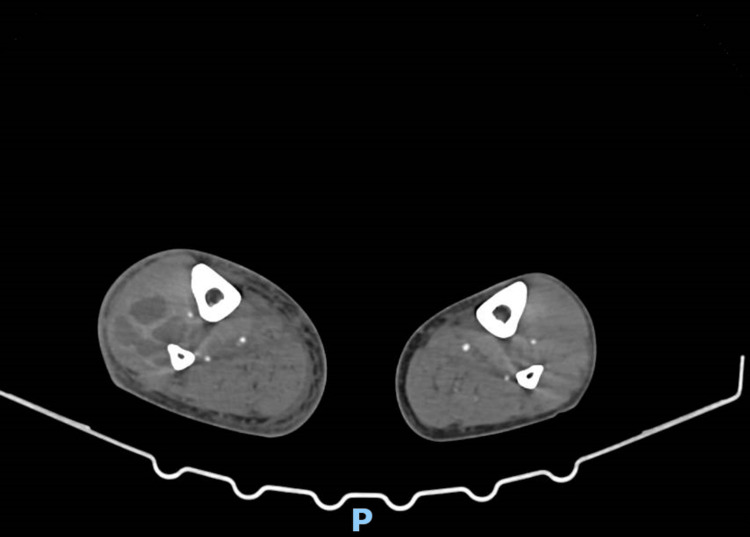
CT scan showing a multiloculated intramuscular collection on the anterior aspect of the proximal third of the left thigh CT scan showing a multiloculated intramuscular collection on the anterior aspect of the proximal third of the left thigh, measuring 5.8 x 4.3 cm in its largest transverse dimensions and 13.3 cm in the longitudinal axis, with slight peripheral enhancement after intravenous contrast administration without active contrast or vascular extravasation (infected hematoma or abcess) and without evidence of active bleeding.

**Figure 3:**
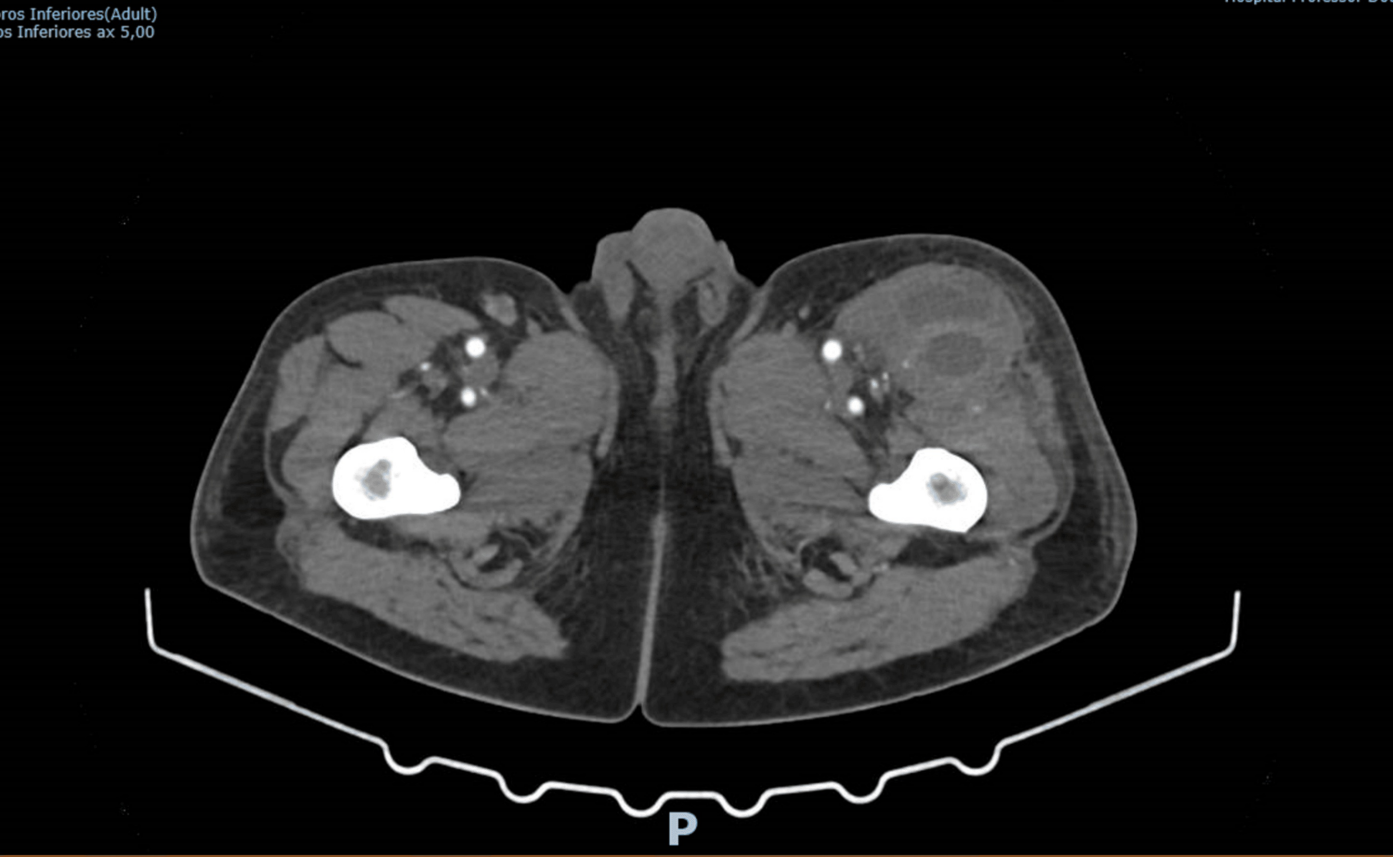
CT scan showing a multiloculated intramuscular collection on the anterolateral aspect of the mid-third of the right leg CT scan showing a multiloculated intramuscular collection on the anterolateral aspect of the mid-third of the right leg, measuring approximately 4.5 x 4.3 cm in its largest transverse dimensions and 13.7 cm in longitudinal length, also showing peripheral enhancement after intravenous contrast administration, suggestive of hematoma versus abscess, without active contrast extravasation.

A 63-year-old male with a history of arterial hypertension and no chronic medication presented to the emergency department with fever, right lower limb edema, erythema, and pain. He denied trauma or known allergies. Laboratory evaluation revealed severe normocytic, normochromic anemia (Hb 5.5 g/dL) with normal white blood cell and platelet counts. C-reactive protein (CRP) was elevated at 14.83 mg/dL. Blood cultures and HIV serology were negative. The patient was admitted with a presumed diagnosis of cellulitis and started on empirical intravenous flucloxacillin. On the second day of hospitalization, he developed two additional painful swellings-in the left thigh (approximately 10 cm) and the right thoracic region. CT angiography revealed three multiloculated intramuscular collections suggestive of hematomas or abscesses, with no signs of active bleeding, as evident in Figures 1-3 [5,6].

**Figure 4:**
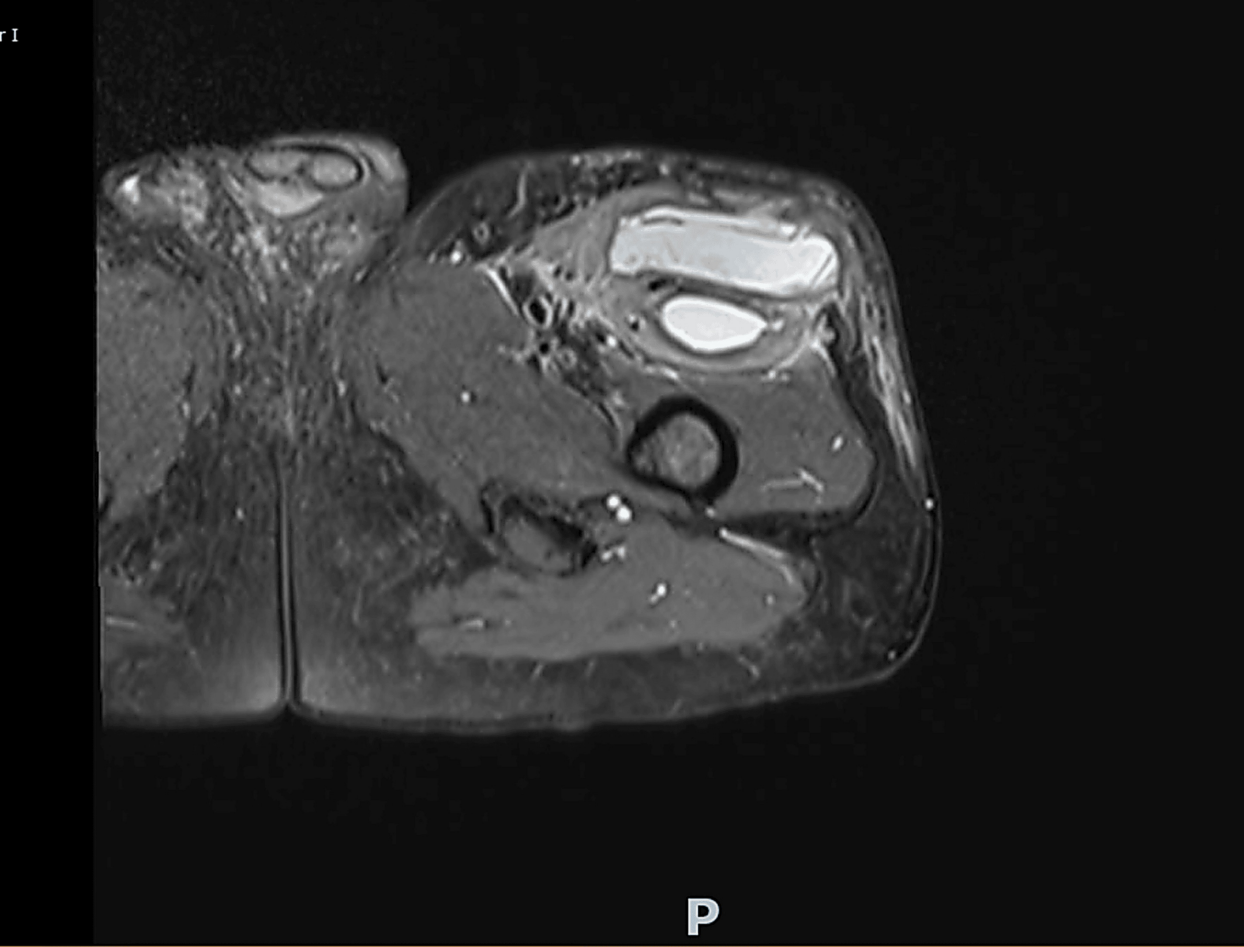
MRI of the left thigh revealing a large multiloculated collection (~15 × 6.5 × 4 cm) with associated myositis in the left thigh muscles, without evidence of soft tissue neoplasm

Given the absence of trauma, anticoagulant use, or thrombocytopenia, spontaneous hematomas were suspected. Investigations excluded vitamin C deficiency and platelet dysfunction. Autoimmune studies, creatine kinase, and electromyography were unremarkable. Percutaneous drainage was performed for all three lesions. MSSA was isolated from the aspirate of one of the lesions, while the others did not yield sufficient material for microbiological analysis. The patient later developed a hypersensitivity reaction to beta-lactams and was switched to clindamycin. Panton-Valentine leukocidin testing was negative. Cytology of drained material revealed polymorphonuclear cells and lymphocytes but no malignant cells. Despite antibiotic therapy and clinical improvement, a residual thigh lesion persisted. MRI revealed a multiseptated collection within the rectus femoris muscle, measuring 15 x 6.5 x 4 cm, without features suggestive of neoplasia (Figure 4).

**Figure 5:**
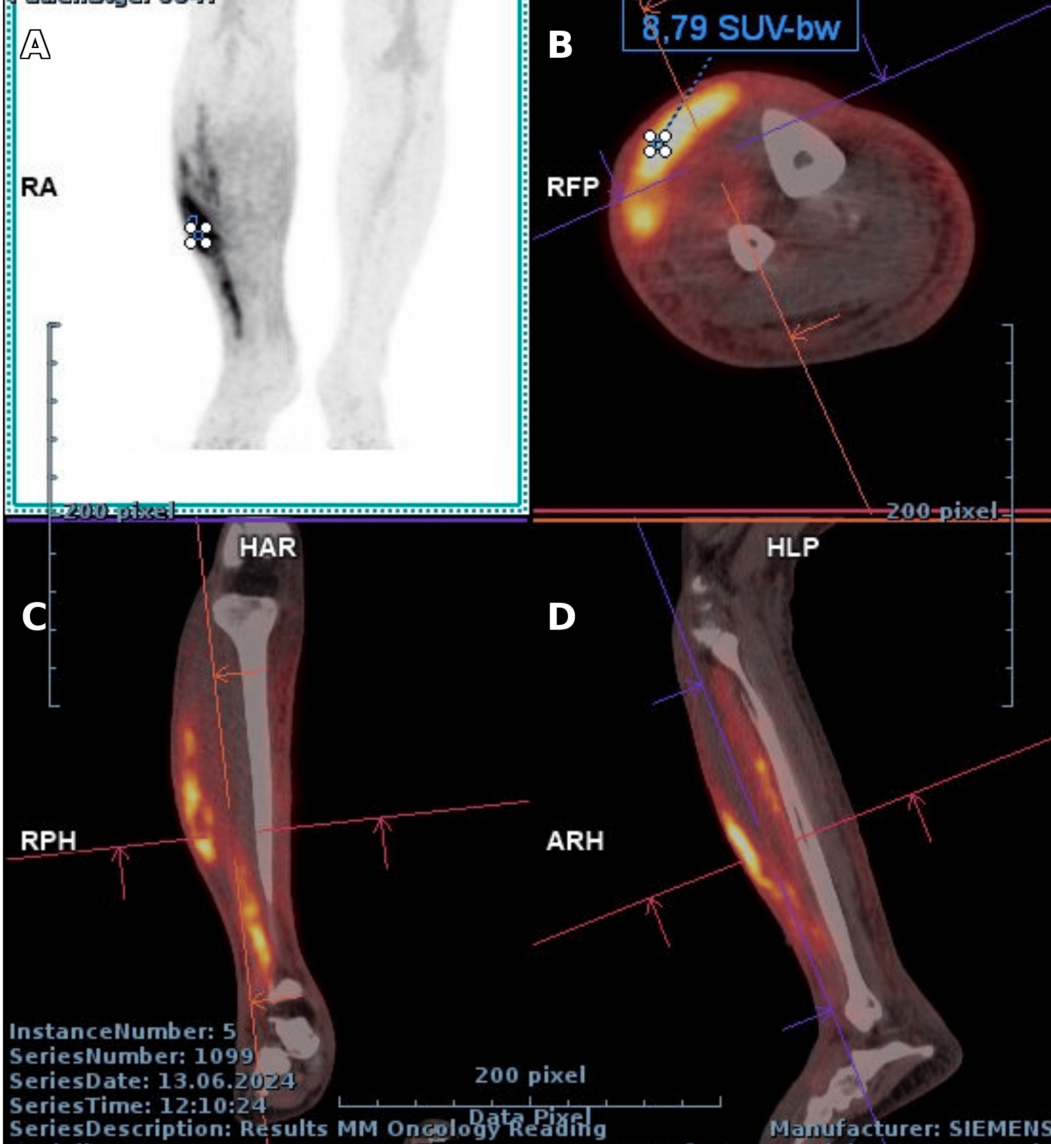
PET/CT of the right lower limb: (A) Maximum intensity projection (MIP) showing hypermetabolic focus in the right leg; (B) Ax slice with SUVmax 8.79; (C) Cor slice confirming longitudinal extension; (D) Sag slice showing distribution along the leg musculature

**Figure 6:**
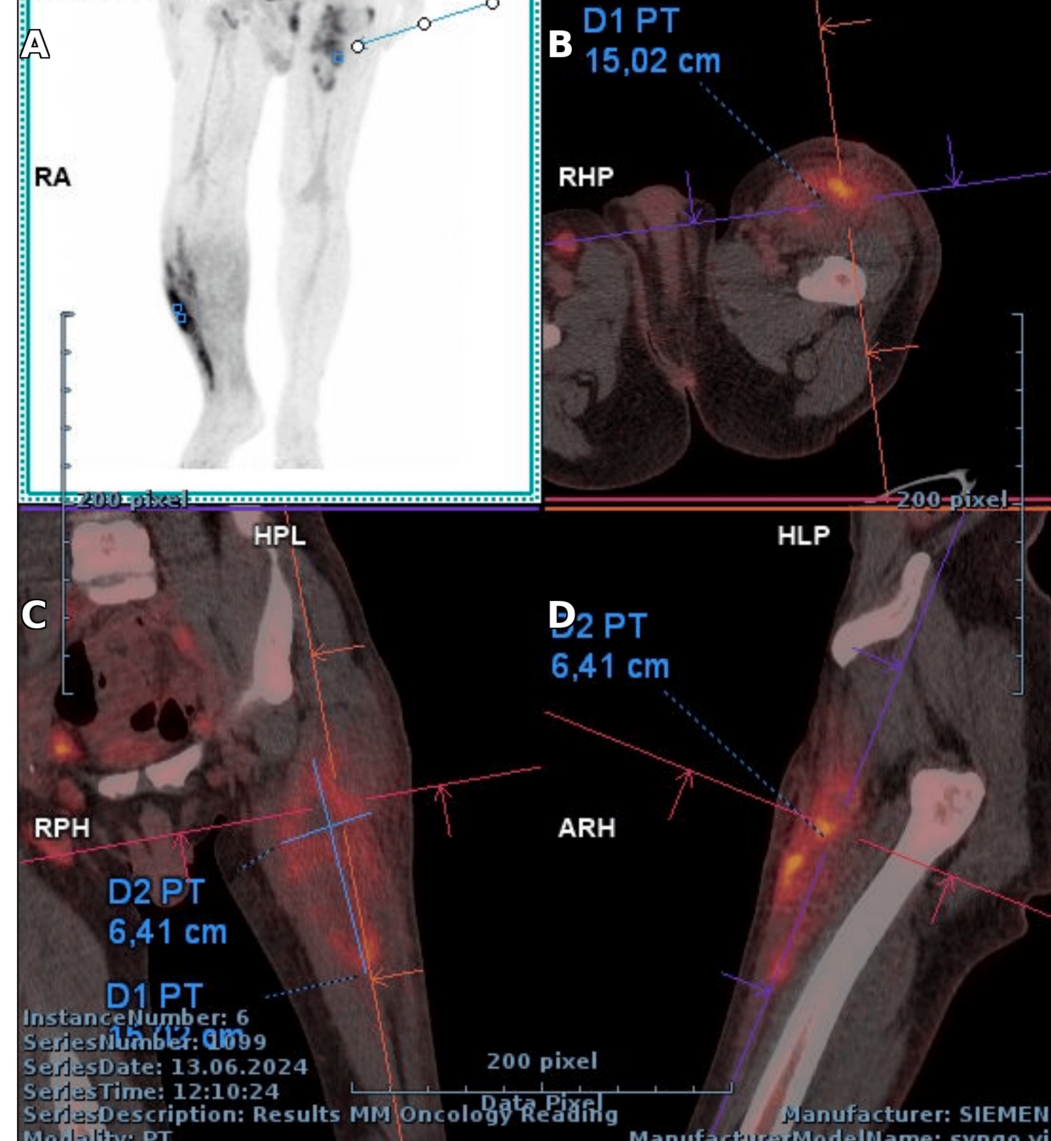
PET/CT of the right lower limb with volumetric analysis: (A) MIP showing two hypermetabolic areas in the right leg and left thigh; (B) Ax slice with lesion 15.02 cm (D1 PT); (C) Cor slice with lesion 6.41 cm (D2 PT); (D) Sag slice confirming both lesions (D1 PT, D2 PT).

**Figure 7:**
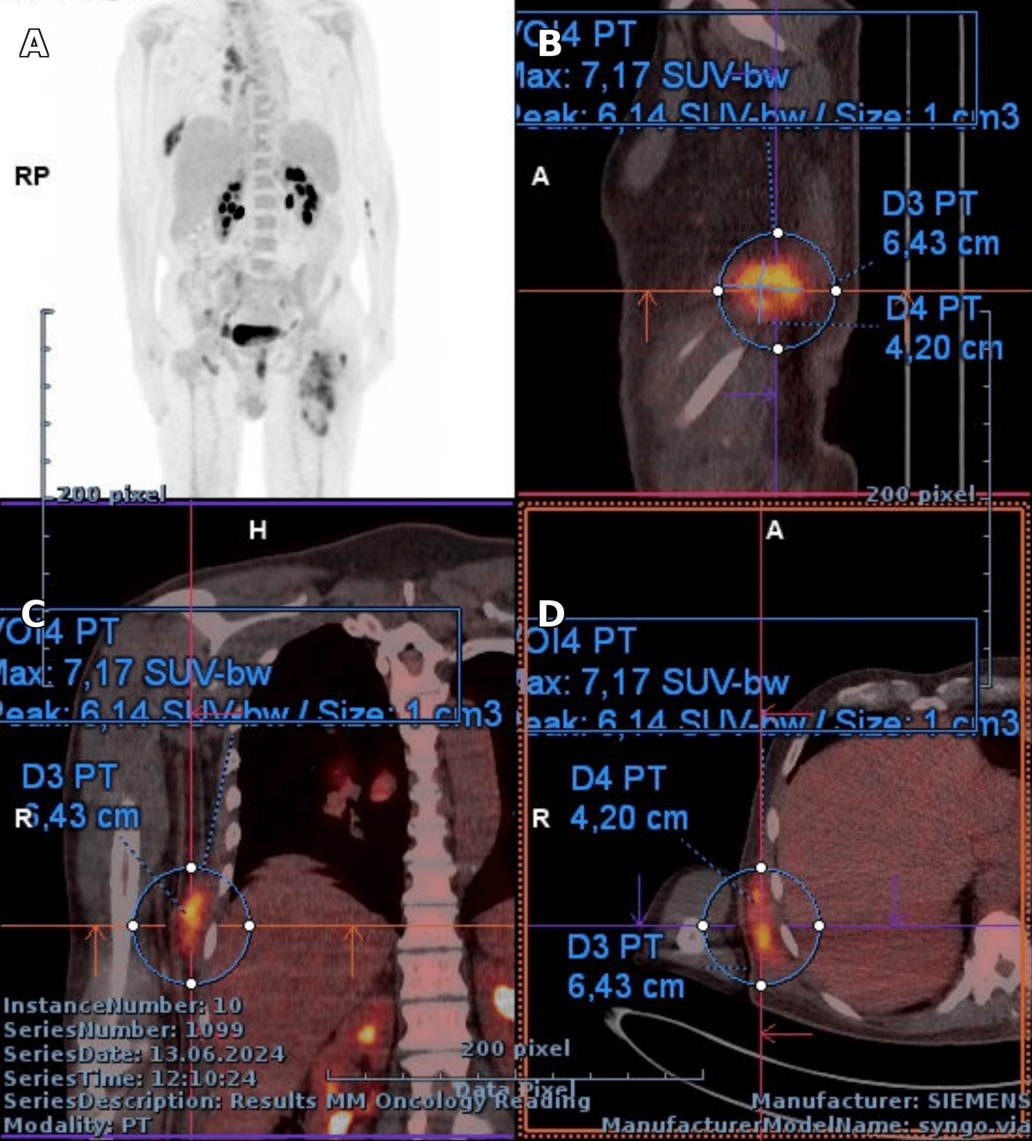
PET/CT of the thorax: (A) MIP showing thoracic hypermetabolic foci; (B) Sag slice with lesion 6.43 cm (D3 PT); (C) Cor slice with SUVmax 7.17 (VOI4 PT); (D) Ax slice confirming lesions 6.43 cm (D3 PT) and 4.20 cm (D4 PT)

PET-CT showed FDG-avid muscular lesions and reactive lymphadenopathy, with no other abnormal uptake, as seen in Figures 5-7 [7,8].

Due to persistent anemia and new-onset neutropenia, bone marrow aspiration and biopsy were performed, revealing a hypercellular marrow (95% cellularity) with trilinear dysplasia and 6% blasts - diagnostic of MDS [9]. A muscle biopsy of the left thigh revealed a cellular infiltrate composed of immature pleomorphic cells with frequent mitoses. Immunohistochemistry was positive for CD34, CD43, CD68, and myeloperoxidase (MPO), consistent with MS [1,2,10]. The patient was referred to a hematology center but initially refused therapy. Over time, the thigh lesion progressed to a malignant ulcer, and the patient developed pancytopenia. A repeat bone marrow biopsy confirmed transformation to acute myelomonocytic leukemia with aberrant expression of CD33, CD13, CD117, and MPO, consistent with AML with myelodysplasia-related changes.

## Discussion

MS is a rare and diagnostically challenging presentation of myeloid neoplasms and has been reported in unusual anatomical locations, even following myeloproliferative disorders such as myelofibrosis [11]. It may present as an isolated finding or in association with systemic disease. This case is notable for its initial presentation mimicking cellulitis and abscess formation, diverting early suspicion from a hematologic origin. The diagnosis of MS requires histopathologic confirmation with immunohistochemical staining for markers such as CD34, CD43, and MPO [1,10]. Imaging studies, including MRI and PET-CT, may assist in identifying lesions but are rarely diagnostic. Cytology from aspirated material may be insufficient to exclude malignancy, as illustrated here [4,7]. Delayed diagnosis in MS, especially when occurring without overt leukemia, may lead to disease progression and worse outcomes [2,8,12]. This case emphasizes the need for early biopsy in unexplained soft tissue lesions, particularly in patients with concurrent cytopenias.

## Conclusions

MS, although rare, should be considered in the differential diagnosis of atypical soft tissue lesions, particularly in the presence of unexplained anemia or neutropenia. As this case demonstrates, misdiagnosis can lead to treatment delays and disease progression. In rare, but clinically significant cases, early biopsy and hematologic evaluation are essential to ensure accurate diagnosis and timely management.
